# Endoscopic ultrasound-guided detection and internal drainage of a pancreato-cutaneous fistula after acute pancreatitis

**DOI:** 10.1055/a-2358-1333

**Published:** 2024-07-26

**Authors:** Ahmad Kattan, Olivier Monneuse, Alice Burgevin, Rosario DʼAlmeida, Gwladys Pointet, Jérôme Rivory, Mathieu Pioche

**Affiliations:** 1 Gastroenterology and Endoscopy Unit, Edouard Herriot Hospital, Lyon, France; 2 Digestive Surgery, Edouard Herriot Hospital, Lyon, France

Pancreatocutaneous fistulas are an uncommon complication of acute pancreatitis 1 2 . Endoscopic management of these fistulas is not straightforward 3 . One approach involves catheterizing the fistula through the skin using a percutaneous drain; however, catheterization of the disconnected pancreatic duct is nearly impossible. Alternatively, pigtail drains can be placed in the peripancreatic collection, if present, but this cavity is often virtual and tends to empty through the cutaneous fistula 4 5 .

We report a case of a 37-year-old patient with severe acute necrotic pancreatitis and multiorgan failure complicated by ischemic colitis. Surgery was performed, including necrosectomy, left colectomy, and placement of a surgical drain in contact with the pancreas. The drain spontaneously dislodged 2 months after surgery. Subsequently, the patient presented with fluid leakage from skin and was admitted to the emergency department. A computed tomography (CT) scan revealed a corporocaudal peripancreatic collection along the former path of the surgical drain. This collection communicated with a left subparietal extraperitoneal collection and a transparietal, closed cutaneous fistulous path in the left hypochondrium ( Fig. 1 ). Immediately before the scheduled endoscopy procedure to drain the collection through the stomach, the fistula reopened, and no collection was observed on endoscopic ultrasound (EUS).

**Fig. 1:**
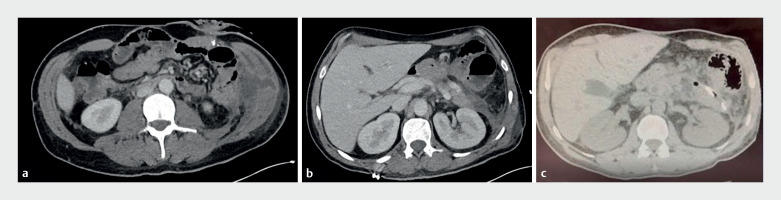
CT images before and after the procedure. **a** Subcutaneous path of the fistula. **b** A 6 × 2-cm-long collection was observed along the fistula path. **c** Regression of peripancreatic collection after the procedure with the pigtail drains in place.

We inserted a guidewire through the skin to place a balloon in the peripancreatic region. This balloon was located using EUS and punctured with a 19-G needle via the transgastric approach ( Fig. 2 , Video 1 ). Next, two guidewires were inserted percutaneously, followed by the passage of a 7-Fr and then 10-Fr cystotome, Subsequently, we inserted two 7-Fr pigtail prostheses to drain both the peripancreatic collection and the fistulous path to the skin, facilitating directed healing of the fistula.

**Fig. 2:**
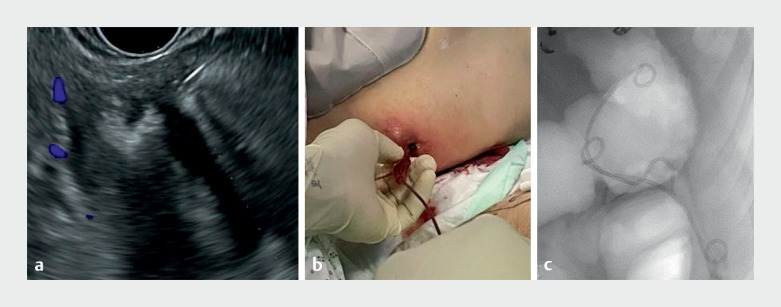
Endoscopic ultrasound (EUS)-guided drainage of a pancreatocutaneous fistula after acute pancreatitis. **a** EUS-guided puncture of the balloon inserted in the peripancreatic region. **b** Appearance of skin opening of the fistula. **c** Endoscopic view of pigtail drains in place.

Endoscopic ultrasound (EUS)-guided detection and puncture of a balloon inserted into a pancreatocutaneous fistula to facilitate delayed scarring.Video 1

After 3 months, the skin opening of the fistula had healed, and a CT scan showed significant regression of the peripancreatic collections with the pigtail drains in place.

Endoscopy_UCTN_Code_TTT_1AS_2AJ

## Notes

Endoscopy E-Videos https://eref.thieme.de/e-videos
*E-Videos* is an open access online section of the journal *Endoscopy* , reporting on interesting cases and new techniques in gastroenterological endoscopy. All papers include a high-quality video and are published with a Creative Commons CC-BY license. Endoscopy E-Videos qualify for HINARI discounts and waivers and eligibility is automatically checked during the submission process. We grant 100% waivers to articles whose corresponding authors are based in Group A countries and 50% waivers to those who are based in Group B countries as classified by Research4Life (see: https://www.research4life.org/access/eligibility/ ). This section has its own submission website at https://mc.manuscriptcentral.com/e-videos .
